# Optimal Resonant Band Demodulation Based on an Improved Correlated Kurtosis and Its Application in Bearing Fault Diagnosis

**DOI:** 10.3390/s17020360

**Published:** 2017-02-13

**Authors:** Xianglong Chen, Bingzhi Zhang, Fuzhou Feng, Pengcheng Jiang

**Affiliations:** 1 Department of Mechanical Engineering, Academy of Armored Forces Engineering, Beijing 100072, China; chenchendeplace@163.com (X.C.); gu_peng88@168.com (P.J.); 2 Beijing Special Vehicle Research Institute, Beijing 100072, China; zhangbingz@foxmail.com

**Keywords:** fault diagnosis, squared envelope spectrum, optimal resonant band demodulation, correlated kurtosis, rolling bearing

## Abstract

The kurtosis-based indexes are usually used to identify the optimal resonant frequency band. However, kurtosis can only describe the strength of transient impulses, which cannot differentiate impulse noises and repetitive transient impulses cyclically generated in bearing vibration signals. As a result, it may lead to inaccurate results in identifying resonant frequency bands, in demodulating fault features and hence in fault diagnosis. In view of those drawbacks, this manuscript redefines the correlated kurtosis based on kurtosis and auto-correlative function, puts forward an improved correlated kurtosis based on squared envelope spectrum of bearing vibration signals. Meanwhile, this manuscript proposes an optimal resonant band demodulation method, which can adaptively determine the optimal resonant frequency band and accurately demodulate transient fault features of rolling bearings, by combining the complex Morlet wavelet filter and the Particle Swarm Optimization algorithm. Analysis of both simulation data and experimental data reveal that the improved correlated kurtosis can effectively remedy the drawbacks of kurtosis-based indexes and the proposed optimal resonant band demodulation is more accurate in identifying the optimal central frequencies and bandwidth of resonant bands. Improved fault diagnosis results in experiment verified the validity and advantage of the proposed method over the traditional kurtosis-based indexes.

## 1. Introduction

Rolling bearings are one of the most common but the most vulnerable parts in rotating mechanical systems. In order to ensure uninterrupted operation and avoid unexpected failures, research attention has been focused on the extraction of weak fault features of rolling bearings which constitute a key factor to condition monitoring and fault diagnosis of rotating mechanical systems [1]. Bearings usually generate wide-band impulses according to their fault frequency and force the bearing system to render an impulse attenuation response when a local fault failure occurs in the inner ring, outer ring, rolling element or cage of a rolling bearing,. As a result, transient impulses in vibration signals of rolling bearings occur cyclically. A popular consensus among the researchers is that the resonant band demodulation can be an effective method in extracting fault features and diagnosing faults of rolling bearings. The key factor of resonant band demodulation is to accurately achieve the central frequency and the bandwith of the optimal resonant frequency band. However, transient features of rolling bearings can be greatly compromised due to the heavy background noise and signal transmission paths. Consequently, it becomes difficult to identify resonant frequency bands, not to mention the accurate diagnosis of such faults by resonant frequency band demodulation [2].

Antoni [3,4] put forward the kurtogram based on spectral kurtosis for the detection of non-stationary transients and their frequency locations. By combining resonant frequency band demodulation, the kurtogram can effectively diagnose faults of rolling bearings. In order to improve the computation performance of the kurtogram, Antoni [5] further built the fast kurtogram by combining iterative segmentation of frequency range such as binary tree and band-pass filters such as short-time Fourier Transform. Taking advantage of the superiority of the fast kurtogram, several in-depth studies have been done in the area of bearing vibration monitoring and fault diagnosis [6,7,8,9,10]. However, the fast kurtogram has two deficiencies. Firstly, kurtosis can only characterize the strength of transient impulses, but it cannot differentiate impulse noises and transient impulses, which are cyclically generated in rolling bearing vibration signals. As a result, it may lead to the inaccurate resonance band identification results, unsatisfactory fault feature demodulation results and misleading rolling bearing fault diagnosis results [11,12,13]. Secondly, the fast kurtogram cannot accurately perfectly determine the central frequencies and bandwidth of rolling bearings’ resonant frequency bands by roughly segmenting frequency ranges [14,15], which may also lead the unsatisfactory demodulation results of fault features. Meanwhile, some artificial intelligence algorithms are also proposed to detect bearing faults [16,17,18].

In view of these deficiencies, Wang [10] proposed an enhanced kurtogram by calculating kurtosis based on the envelope spectrum of the wavelet package transform filtered signal, which performs well in determining resonance bands, and in demodulating the fault features of rolling bearings. Barszcz [11] proposed the protrugram method based on the kurtosis of the envelope spectrum and narrowband demodulation to select optimal resonant frequency band and to detect transient impulses with smaller rolling bearing vibration signal signal-to-noise ratios. The protrugram shows a superior detection ability of modulated signals in the presence of higher noise than in the case of the fast kurtogram. Enlightened by thermodynamics and entropic uncertainty principle. Antoni et al. [13] proposed the squared envelope infogram (SE infogram) and the squared envelope spectrum infogram (SES infogram) by measuring the negentropy of the squared envelope and the squared envelope spectrum of the short-time Fourier Transform filtered signal, respectively. The SES infogram can effectively provide new information about repetitive transients and locate the resonant frequency bands. Experimental results demonstrate the superiority of SE infogram and SES infogram in selecting resonant bands and demodulating bearing fault features in comparison to the fast kurtogram. Furthermore, Li et al. [14] proposed an optimal demodulation of bearing vibration signals by combining criterion fusion and bottom-up segmentation of the spectral sequence, which effectively determined the optimum resonant frequency band of rolling bearings and improved the robustness in identifying the optimal resonant central frequency. Tse et al. [12] proposed the sparsogram, which is constructed using the sparsity measurements of wavelet packet coefficients’ envelope power spectra at different decomposition depths. The sparsogram effectively locates the resonant bands and shows the superior capability in bearing fault diagnosis by comparison studies with kurtosis, smoothness index and Shannon entropy. Furthermore, Tse et al. [15] further proposed the automatic selection of a perfectly resonant frequency band by combining a complex Morlet wavelet filter and a genetic algorithm to enhance the sparsogram for fault feature demodulation of rolling bearings.

The aforementioned review of related studies indicates that the identification quality of resonant frequency bands depends on the construction of criterion used to detect transient features and on the construction of a band-pass filter used to segment the frequency band. Among them, the construction of criteria used to detect transient features is the key factor to determine the quality of identified resonant frequency bands.

Taking advantage of the periodical features of transient impulses, McDonald et al. [19] proposed the correlated kurtosis to detect repetitive transient impulses. As the kurtosis can only characterize the strength of transient impulses, which cannot differentiate impulse noises and repetitive transient impulses, the correlated kurtosis then becomes a potential substitution criterion for kurtosis-based indexes in detecting repetitive transients. However, the calculation of correlated kurtosis is performed on periodical signals, and previous research results have proved that repetitive transients of rolling bearing are not periodic, but rather are cyclostationary [13]. As a result, the calculation of correlated kurtosis on bearing vibration signals may lack an essential theoretical basis. Besides, the correlated kurtosis proposed by McDonald is only a construction form, which calls for a clear physical definition.

To address these issues, the manuscript redefines the correlated kurtosis, on the basis of kurtosis and auto-correlative function, and puts forward an improved correlated kurtosis based on the squared envelope spectrum of rolling bearing vibration signals. Meanwhile, this manuscript proposes an optimal resonant frequency band demodulation by combining the complex Morlet wavelet band-pass filter and the Particle Swarm Optimization algorithm, which can adaptively identify the optimal resonant frequency bands and demodulate transient features of rolling bearings.

The rest of the manuscript is organized as follows: the related theoretical background is presented in Section 2, the methods proposed by the authors are discussed in Section 3, the simulation analysis and test applications are presented in Section 4, and our conclusions in Section 5.

## 2. Kurtosis and Correlated Kurtosis

### 2.1. Kurtosis

As we know, kurtosis can represent the characteristics of signals around their mean value and characterize the strength of transient impulses. Given a zero mean signals, kurtosis can be defined as the ratio among the fourth central moment of the signal to the squared second central moment of the signal [20]. Given a zero-mean filtered signal $X_{l,h}$, one can obtain its analytic signal ${\tilde{X}}_{l,h}$ as follows:

(1) $${\tilde{X}}_{l,h}=X_{l,h}+j\cdot \mathrm{Hilbert}(X_{l,h}),$$

where *j* is the imaginary number, Hilbert(·) the Hilbert Transform function, *h* and *l* are respectively the upper and lower cut-off frequency of the band-pass filter $f_{l}^{h}$.

Then the kurtosis can be expressed as:

(2) $$\mathrm{Kurtosis}({\tilde{X}}_{l,h})=\frac{E({\left|{\tilde{X}}_{l,h}\right|}^{4})}{{\left[E({\left|{\tilde{X}}_{l,h}\right|}^{2})\right]}^{2}},$$

where E(·) is the mathematical expectation operator.

Taking a step further, the corresponding transient impulses can lead to an instantaneous energy fluctuation in vibration signals when a rolling bearing catches local faults. The squared envelope signal can represent the instantaneous energy fluctuation of the signal [13]. Given a zero-mean filtered signal ${\tilde{X}}_{l,h}$, one can express its squared envelope signal $\mathrm{SE}({\tilde{X}}_{l,h})$ as follows:

(3) $$\mathrm{SE}({\tilde{X}}_{l,h})={\left|{\tilde{X}}_{l,h}\right|}^{2}={\left|X_{l,h}+j\cdot \mathrm{Hilbert}(X_{l,h})\right|}^{2},$$

the variance of $\mathrm{SE}({\tilde{X}}_{l,h})$ can be expressed as:

(4) $$D\left[\mathrm{SE}({\tilde{X}}_{l,h})\right]=E{\left[\mathrm{SE}({\tilde{X}}_{l,h})\right]}^{2}-{\left\{E\left[\mathrm{SE}({\tilde{X}}_{l,h})\right]\right\}}^{2},$$

the normalized version of $D\left[\mathrm{SE}({\tilde{X}}_{l,h})\right]$ can be expressed as:

(5) $$d\left[\mathrm{SE}({\tilde{X}}_{l,h})\right]=\frac{E{\left[\mathrm{SE}({\tilde{X}}_{l,h})\right]}^{2}}{{\left\{E\left[\mathrm{SE}({\tilde{X}}_{l,h})\right]\right\}}^{2}}-1.$$

Substituting Equation (5) into Equation (1), a new expression of kurtosis can be expressed as:

(6) $$\mathrm{Kurtosis}({\tilde{X}}_{l,h})=d\left[\mathrm{SE}({\tilde{X}}_{l,h})\right]+1.$$

As we know, the resonant frequency band filtered signal contains prominent repetitive transient fault impulses when a rolling bearing records local faults. Those repetitive transient fault impulses can lead to instantaneous energy fluctuations in the filtered signal and amplify the normalized variance in Equation (6). As a result, the resonant frequency band filtered signals usually have a high kurtosis value, and resonant frequency band demodulation based on kurtosis citation can be effective in detecting transient fault impulses and diagnosing faults of rolling bearings, such as the fast kurtogram. However, the vibration signals of rolling bearings always contain many other components, which may be useless for bearing diagnosis, such as discrete impulsive noises. Discrete impulsive noises also can give rise to an instantaneous energy fluctuation of signals. The filtered signal, which may not contain repetitive transient impulses but rather contain discrete impulsive noises, can also have a high kurtosis value. As a result, the kurtosis can only characterize the strength of impulses, but it cannot effectively differentiate impulsive noise and repetitive transient fault impulses. As a result, the kurtosis may lead to the inaccurate identification results of resonant bands, the unsatisfactory demodulation results of fault features and the misleading fault diagnosis results of rolling bearings.

### 2.2. Correlated Kurtosis

Thanks to the superiority of kurtosis in characterizing transient impulses, it has been widely used in fault diagnosis of rolling bearings [6]. However, McDonald et al. [19] observed that when compared with a signal containing repetitive periodicity of impulses, a signal with a single impulse would generate a higher kurtosis value. He then concluded that the kurtosis is more effective in detecting a single impulse than repetitive impulses. In reality, rotating machines’ vibration signals usually contain repetitive impulses produced by mechanical faults, rather than a single impulse possibly produced by heavy background noise. Consequently, the kurtosis interfered by heavy noise may correspond with incorrect transient fault features. To address the problem, McDonald et al. [19] proposed the correlated kurtosis (CK) with the help of the impulse periodicity existing in signals.

The CK of zero-mean filtered signal ${\tilde{X}}_{l,h}$ can be formally constructed as [19]:

(7) $$\mathrm{CK}({\tilde{X}}_{l,h},T)=\frac{\sum_{n=1}^{N}{\left[\left|{\tilde{x}}_{l,h}^{*}(n)\right|\cdot \left|{\tilde{x}}_{l,h}(n-T)\right|\right]}^{2}}{{\left[\sum_{n=1}^{N}{\left|{\tilde{x}}_{l,h}(n)\right|}^{2}\right]}^{2}},$$

where *T* is the periodicity of impulses, ${\tilde{x}}_{l,h}(n)$ the *n*th element of the filtered signal ${\tilde{X}}_{l,h}$.

McDonald et al. [19] later verified that CK hit a maximum at a certain period as opposed to the kurtosis which is likely to reach a maximum with a single impulse. CK takes advantage of the periodical features of transient impulses as well as the impulse-like vibration behavior associated with most types of faults. Therefore, it is more effective in detecting signal repetitive transients. Owing to its claimed superiority, some further studies have been done in bearing fault diagnosis and degradation analysis [21,22].

## 3. The Proposed Optimal Resonant Band Demodulation Method Based on an Improved Correlated Kurtosis

In this section, the authors firstly redefine the correlated kurtosis and then propose the improved correlated kurtosis based on squared envelope spectrum; then propose an optimal resonant band demodulation, by combining the complex Morlet wavelet filter and the Particle Swarm Optimization algorithm, to adaptively identify the optimal resonant frequency bands and demodulate the transient features of rolling bearings.

### 3.1. Redefinition of Correlated Kurtosis

Taking advantage of the periodical features of transient impulses, the correlated kurtosis is a potential substitution criterion for kurtosis-based indexes in detecting repetitive transient impulses. However, the correlated kurtosis proposed by McDonald is only a construction form, which calls for a clear physical definition. In this manuscript, the authors redefine the correlated kurtosis as follows:

As the auto-correlative function of the filtered signal $\tilde{X}$ can be expressed as:

(8) $$r_{\tilde{X}}(m)=E\left[{\tilde{x}}^{*}(n)\times \tilde{x}(n+m)\right],$$

where *m* is the time delay coefficient. Substituting Equations (3) and (8) into Equation (2), a new expression of the kurtosis can be obtained as:

(9) $$\mathrm{Kurtosis}({\tilde{X}}_{l,h})\;=\frac{r_{\mathrm{SE}}(0)}{{\left[r_{{\tilde{X}}_{l,h}}(0)\right]}^{2}},$$

where $r_{{\tilde{X}}_{l,h}}(0)$ is the auto-correlative function of ${\tilde{X}}_{l,h}$ when *m* equals to zero. And $r_{SE}(0)$ is the auto-correlative function of squared envelope signal $\mathrm{SE}({\tilde{X}}_{l,h})$ when *m* equals to zero.

The auto-correlative function can be used to detect hidden periodicity of signals. If the periodicity of a finite length signal $\tilde{X}$ is *T*, the auto-correlative function $r_{\tilde{X}}(m)$ of $\tilde{X}$ will also present the periodicity of *T*, and $r_{\tilde{X}}(m)$ contains gradual attenuation peak values only when m=0,T,2T....

Enlightened by the characteristic of auto-correlation function and combining Equation (9), the authors redefine the correlated kurtosis as the ratio between $r_{SE}(T)$ to $r_{{\tilde{X}}_{l,h}}{\left(0\right)}^{2}$. Given a zero-mean filtered signal ${\tilde{X}}_{l,h}$, the redefined correlated kurtosis can be expressed as:

(10) $$\mathrm{ReCK}({\tilde{X}}_{l,h},T)=\frac{r_{\mathrm{SE}}(T)}{{\left[r_{{\tilde{X}}_{l,h}}(0)\right]}^{2}}=N\times \frac{\sum_{n=1}^{N}{\left[\left|\tilde{x}(n)\right|\times \left|\tilde{x}(n+T)\right|\right]}^{2}}{{\left[\sum_{n=1}^{N}{\left|\tilde{x}(n)\right|}^{2}\right]}^{2}},$$

where *N* is the length of the filtered signal, *T* the periodicity of transient impulses, $r_{SE}(T)$ the auto-correlative function of the squared envelope signal when *m* equals to *T*, and $r_{{\tilde{X}}_{l,h}}(0)$ the auto-correlative function of the filtered signal when *m* equals to zero.

In comparison with the correlated kurtosis proposed by McDonald in Equation (7), the redefined correlated kurtosis in Equation (10) has two distinctions. One is that the redefined correlated kurtosis utilizes signal length *N* as its correction factor and the other is that the computation of the redefined correlated kurtosis moves *T* points of the signal to right, instead of to left. Moreover, inheriting characteristics of auto-correlative function and kurtosis, the correlated kurtosis redefined in this manuscript can not only reduce impacts of impulsive noises and uncorrelated transient impulses on the detection of repetitive transient fault impulses, but also overcome the drawbacks of the kurtosis-based indexes. As a result, the redefined correlated kurtosis can be a potential substitution criterion for kurtosis-based indexes in detecting repetitive transient fault features.

### 3.2. An Improved Correlated Kurtosis

Unlike the kurtosis, the correlated kurtosis both in Equations (7) and (10) is defined on the basis of periodic signals. However, according to cyclostationary analysis, bearing vibration signals have typically some second-order cyclostationary characteristics, repetitive transient fault features of bearing vibration signals are not periodic, but rather cyclostationary, which means the instantaneous energy fluctuates rather than their waveform being periodic [13]. As a result, applying the correlated kurtosis directly on bearing vibration signal may lack in necessary theoretical foundations. To overcome this weakness of correlated kurtosis, the authors observe the frequency spectrum of instantaneous energy fluctuations signal, namely the squared envelope spectrum (SES) of bearing vibration signal, which can be expressed as:

(11) $$\mathrm{SES}(f)=\mathrm{DFT}\left[\mathrm{SE}({\tilde{X}}_{l,h})\right]=\sum_{i\in \mathbb{Z}}\mathrm{SES}(f,k)\delta (f-k\times f_{\mathrm{fault}}),$$

where $f_{\mathrm{fault}}$ is the fault feature frequency of rolling bearing, SES(f,k) the *k*th spectrum line of SES, and δ the unit-impulse function.

It can be seen from Equation (11) that the fault feature frequency $f_{\mathrm{fault}}$ and its harmonics are periodically distributed on frequency axis of SES, and the periodicity of those harmonics corresponds with the fault feature frequency. By performing the computation of the redefined correlated kurtosis on SES, the correlated kurtosis may remedy the weakness. Then, this manuscript proposes an improved correlated kurtosis on the basis of the redefined correlated kurtosis in Equation (10) and SES, which can be expressed as:

(12) $$\mathrm{SESCK}({\tilde{X}}_{l,h},f_{\mathrm{fault}})=(h-l\;+\;1)\times \frac{\sum_{f=0}^{h-l}{\left[\mathrm{SES}(f)\times \mathrm{SES}(f+f_{\mathrm{fault}})\right]}^{2}}{{\left[\sum_{f=0}^{h-l}\mathrm{SES}{\left(f\right)}^{2}\right]}^{2}}$$

where *h* and *l* are upper and lower cut-off frequency of band-pass filter $f_{l}^{h}$.

Comparing Equation (10) with Equation (12), one can learn that Equation (12) is the implementation of Equation (10) on SES. The improved correlated kurtosis can approach a higher value when the SES of filtered signal contains higher peak values of the fault feature frequency, more harmonics, and wider filtered bandwidth. On the contrary, the improved correlated kurtosis approaches an especially small value when the SES contains lower peak values of the fault feature frequency, less harmonics, and narrower filtered bandwidth, or even doesn’t contain any fault feature frequency, nor its harmonics. As a result, it also can overcome the drawbacks of the kurtosis-based indexes.

### 3.3. Performance Comparison

**Figure 1:**
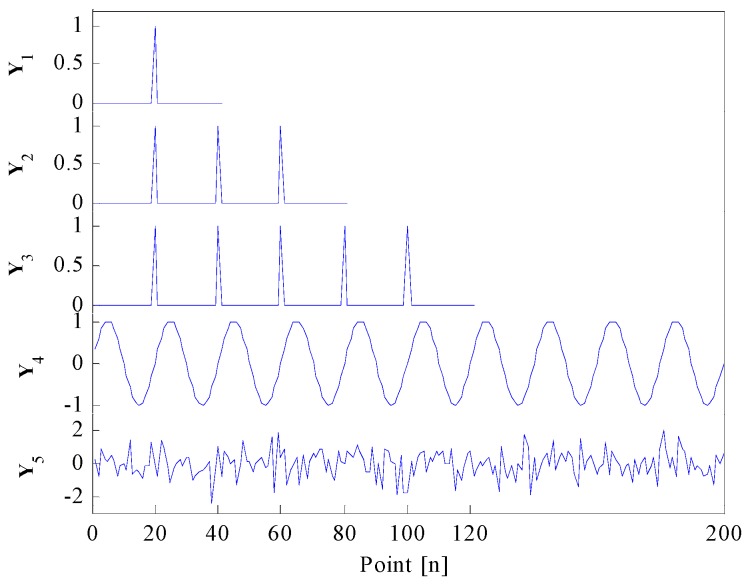
Testing signals.

In previous subsections, the authors have redefined the correlated kurtosis and proposed an improved correlated kurtosis based on SES. In order to verify the superiority of the redefined correlated kurtosis and the improved correlated kurtosis against the kurtosis, spectral kurtosis and the correlated kurtosis originated by McDonald, several discrete testing signals, shown in Figure 1, are constructed as follows:

Signal **Y**_1_: a unit-impulse function *δ*(20) and its length equals to 40.
Signal **Y**_2_: a Dirac comb function composed by unit-impulse functions *δ*(20), *δ*(40) and *δ*(60), its length equals to 80.
Signal **Y**_3_: a Dirac comb function composed by unit-impulse functions *δ*(20), *δ*(40), *δ*(60), *δ*(80) and *δ*(100), its length equals to 120.
Signal **Y**_4_: a sine function and its length equals to 200.
Signal **Y**_5_: a Gaussian noise function and its length equals to 200.

As is mentioned in references [11,13], a Dirac comb stands as an idealization of a series of repetitive transients and a single impulse is a particular instance of a Dirac comb, though they are unlikely to occur in practice. The characteristic of repetitive transients in time is also a series harmonic in SES. Fortunately, a Dirac comb has the same structure in both time domain and SES. Therefore, Dirac comb functions can be utilized herein to compare performances of different indexes in detecting repetitive transients.

As the kurtosis, the original correlated kurtosis and the redefined correlated kurtosis are all defined in the time domain, in order to compare their performances in detecting repetitive transient impulses, firstly, treating testing signals in Figure 1 as time singles. The results calculated by the kurtosis, the correlated kurtosis originated by McDonald and the redefined correlated kurtosis are shown in Table 1.

From Table 1, one can learn that, the results of **Y**_1_, **Y**_2_ and **Y**_3_ calculated by the kurtosis have the same value, even if **Y**_1_, **Y**_2_ and **Y**_3_ contain different quantities of repetitive transient impulses. As a result, the kurtosis in the time domain cannot differentiate a single impulse and a series of repetitive impulses, the latter of which is usually believed to be more helpful in fault diagnosis. The result of **Y**_2_ calculated by the correlated kurtosis originated by McDonald is larger than other signals, especially larger than **Y**_3_, which is the optimal repetitive impulses with more obvious periodicity than **Y**_2_. Such is the case, the correlated kurtosis originated by McDonald might fail in identifying the optimal filtered signal which contains more repetitive impulses. Finally, the result of **Y**_3_ calculated by the redefined correlated kurtosis is larger than other signals. Thus, the redefined correlated kurtosis claims the superiority over other methods in identifying the optimal filtered signal which contains more repetitive impulses.

As a Dirac comb has the same structure in both time domain and SES, treating testing signals **Y**_1_, **Y**_2_ and **Y**_3_ in Figure 1 as SES of themselves. Then computation results of **Y**_1_, **Y**_2_ and **Y**_3_ calculated by spectral kurtosis and the improved correlated kurtosis are shown in Table 2. Due to the same construction of kurtosis and spectral kurtosis, the same construction of the redefined correlated kurtosis and the improved correlated kurtosis based on SES. The computation results in Table 2 have same values as is shown in Table 1 when treating testing signals as their SES. Then, one can come a same conclusion that, the spectral kurtosis cannot differentiate a single impulsive frequency and a series of repetitive impulse harmonics, the improved correlated kurtosis can identify the optimal filtered frequency band, which contains optimal repetitive impulsive harmonics in SES.

As is mentioned above, the redefined correlated kurtosis can detect the optimal repetitive transient impulses in time domain and the improved correlated kurtosis can detect the optimal repetitive impulsive harmonics in SES. Consequently, the performance comparison can verify the superiority of the redefined correlated kurtosis and the improved correlated kurtosis.

### 3.4. The Optimal Resonant Band Demodulation

The resonant frequency band demodulation is an effective method for extracting fault features and diagnosing faults of rolling bearings, and the key factor of resonant frequency band demodulation is to accurately identify the central frequency and the bandwith of the optimal resonant frequency band. However, the identification quality of resonant band depends on the construction of criterion used in detecting transient features and on the construction of band-pass filter used in segmenting frequency band.

Firstly, as is demonstrated in previous subsections, the improved correlated kurtosis on the basis of SES can not only reduce the impacts of impulse noises in detecting repetitive transient fault features, but also can remedy the drawbacks of the kurtosis-based indexes. Thus, the improved correlated kurtosis can be a substitution criterion for kurtosis-based indexes in detecting repetitive transient fault features.

Secondly, the complex Morlet wavelet is a kind of band-pass filter. Its wavelet function contains exponential attenuation components, which correspond with transient characteristics of rolling bearing faults. The wavelet function of complex Morlet wavelet φ(t) can be expressed as [23,24,25,26]:

(13) $$\varphi (t)=\frac{\alpha }{\sqrt{\pi }}\exp(-\alpha^{2}\pi^{2})\exp(j2\pi f_{c}t),$$

its Fourier Transform ψ(f) can be expressed as:

(14) $$\psi (f)=\psi^{*}(f)=\exp\left[-\frac{\pi^{2}}{\delta^{2}}{\left(f-f_{c}\right)}^{2}\right],$$

where *α* is the envelope factor and $f_{c}$ the central frequency of the complex Morlet wavelet.

**Figure 2:**
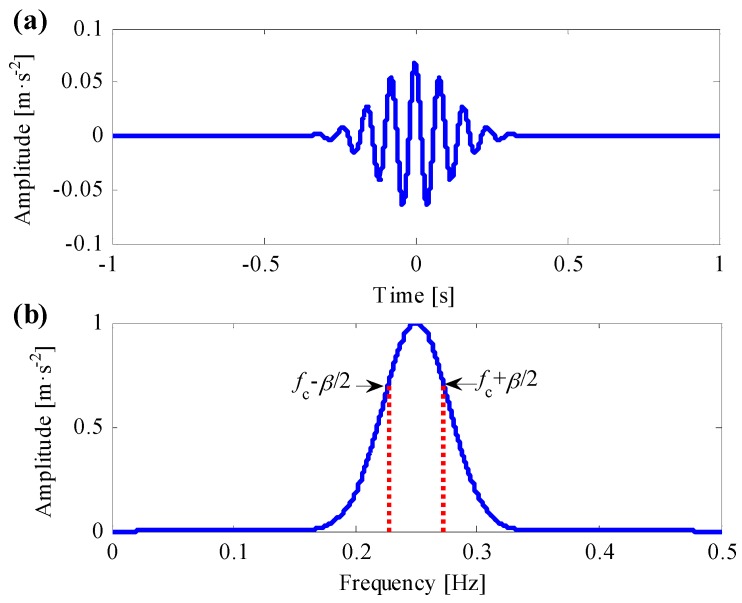
(**a**) Time waveform of the Morlet wavelet; (**b**) frequency waveform of the Morlet wavelet.

Taking normalized central frequency $f_{c}=0.25\;Hz$ and envelope factor α=0.06 Hz as an example, the Morlet wavelet waveform both in time and frequency domain are shown in Figure 2. The filtered bandwidth is defined as *β*, and it equals to the frequency range between the upper cut-off frequency and lower cut-off frequency, which correspond with the $\sqrt{2}/2$ times maximum Morlet wavelet amplitude in frequency domain [26], as shown in Figure 2b. As a result, the filtered range of the complex Morlet wavelet is from $f_{c}-\beta /2$ to $f_{c}+\beta /2$, and $\beta =\alpha \sqrt{2\ln2}/\pi$.

Then the complex Morlet wavelet filtered signal can be expressed as:

(15) $$W_{x}(f_{c},\beta )=F^{-1}\left[X(f)\psi^{*}(f)\right],$$

where $F^{-1}$ is the inverse Fourier Transform function, X(f) the Fourier Transform of signal x(t) and $W_{x}(f_{c},\beta )$ the filtered signal of x(t) by the complex Morlet wavelet filter.

Finally, an optimal resonant band demodulation method, by combining the improved correlated kurtosis and the complex Morlet wavelet filter, is proposed for the purpose of diagnosing faults of rolling bearings in this manuscript. Coupled with the Particle Swarm Optimization algorithm, the proposed method can adaptively identify the optimal resonant bands and demodulate transient features of rolling bearings.

**Figure 3:**
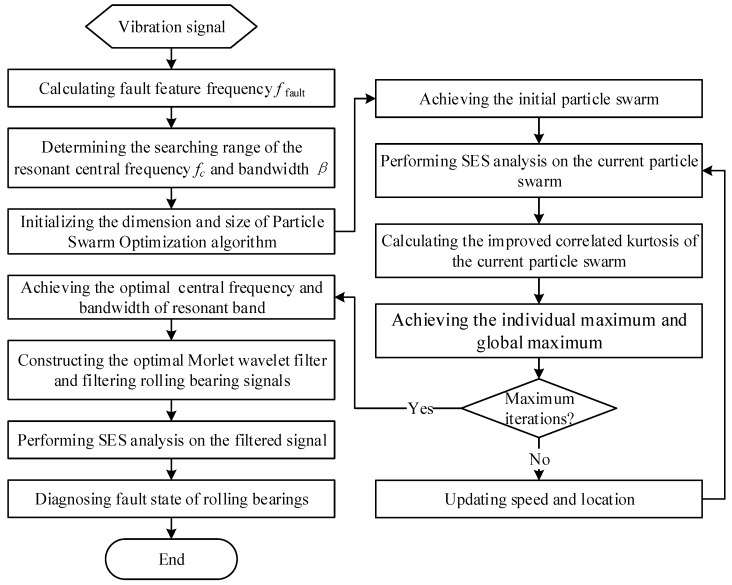
Flowchart of the proposed optimal resonant band demodulation method.

The flowchart of the proposed method is shown in Figure 3, and the detailed steps are as follows:

(1) Calculating the fault feature frequency according to the geometric parameters of the rolling bearing and operation conditions of the mechanical system.
(2) Determining the searching range of the resonant bandwidth. As the band-pass filtered bandwidth should be greater than three times of the fault feature frequency, the searching range of the resonant bandwidth can be set as $3\times f_{\mathrm{fault}}\leq \beta \leq 10\times f_{\mathrm{fault}}$.
(3) Determining the searching range of the resonant central frequency. As the filtered range of the Morlet wavelet filter is $\left[f_{c}-\beta /2,f_{c}+\beta /2\right]$, the searching range of the resonant central frequency can be set as $\beta_{\min}/2\leq f_{c}\leq f_{s}/2-\beta_{\min}/2$, where $f_{s}$ represents the sampling frequency.
(4) Initializing the Particle Swarm Optimization algorithm. The dimension of the particle swarm is set as 2, the size of the particle swarm is set as 30. The initial particle swarm can be achieved in line with the searching settings in step (2) and step (3).
(5) Performing SES analysis on the initial particle swarm, calculating their values of the improved correlated kurtosis and achieving the individual maximum and global maximum.
(6) Updating speed and location of the particle swarm iteratively until the maximum iterations. Achieving the optimal resonant central frequency $f_{c\_\mathrm{opt}}$ and bandwidth $\beta_{\mathrm{opt}}$.
(7) Setting the central frequency of the optimal complex Morlet wavelet filter as $f_{c\_\mathrm{opt}}$, the bandwidth of the optimal complex Morlet wavelet filter as $\beta_{\mathrm{opt}}$. Achieving the optimal resonant band filtered signal.
(8) Performing the SES of the optimal resonant band filtered signal and diagnosing faults of rolling bearings.

## 4. Analysis

### 4.1. Simulation Analysis

A numerical simulation model of rolling bearing vibration is used to construct bearing failure simulation signals to verify the validity of the proposed method. The numerical simulation model is expressed as follows:

(16) $$\left\{ \begin{array}{l} x(t)=s(t)+n(t)\\ s(t)=\sum_{j}h(t-j\times T-\tau_{i})+p(t+t_{1})+p(t+t_{2})\\ h(t)=A_{0}\exp(-Ct)\cos(2\pi f_{n}t)\\ p(t)=M_{0}\exp(-Dt)\cos(2\pi f_{m}t) \end{array}\right.$$

where the rolling bearing simulation signal x(t) includes impulse series s(t) and noise signal n(t). *T* is the average period of impulse series; $f_{i}$ is the impulsive feature frequency which equals to the reciprocal of *T* and set to 100 Hz; $\tau_{i}$ is the tiny random fluctuation of the *i*-th impulse and τ∼N(0,0.05T); *C* is the damping coefficient which equals to 900; $f_{n}$ is the resonant frequency of the simulated rolling bearing system which equals to 4000 Hz; $A_{0}$ is the vibration amplitude which equals to 1; p(t) is additional impulsive noise; *D* is the damping coefficient of the impulsive noise which equals to 600; $f_{m}$ is the resonant frequency of the impulsive noise which equals to 2500 Hz; and $M_{0}$ is the vibration amplitude of the impulsive noise which equals to 5. Two impulse noise components are added to s(t) at the moment of $t_{1}=0.125\;s$ and $t_{1}=0.225\;s$, respectively. The noise signal n(t) is white Gaussian noise with noise variation equaling to 1. The sampling frequency of simulation signal is set to 12,800 Hz while the sampling points are set to 6400.

**Figure 4:**
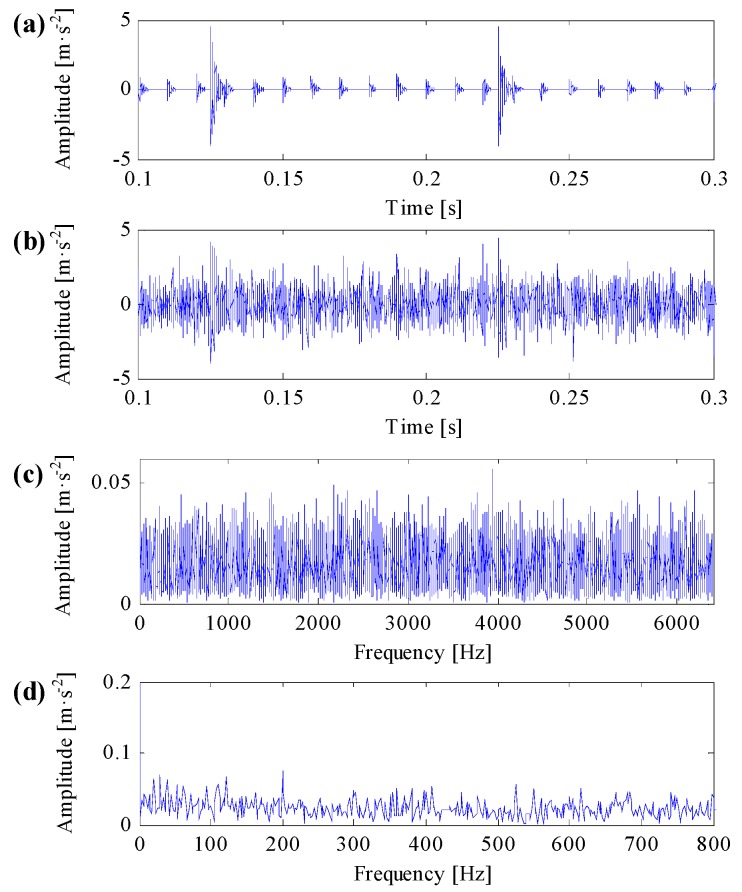
(**a**) Repetitive transient impulses and impulsive noises in the simulation signal; (**b**) the simulation signal with white Gaussian noise; (**c**) the spectrum of the simulation signal; (**d**) the SES of the simulation signal.

In line with Equation (16), a simulation signal is generated as is shown in Figure 4. The simulation signal only includes impulsive series and impulsive noise components is shown in Figure 4a. The repetitive transient impulsive series, additional impulsive noise components and their transient features are distinct in the signal. When the simulation signal includes noise signal and noise variation equals to 1, the signal-to-noise ratio of the simulation signal is −14.9 dB. And the simulation signal waveform both in time domain and in frequency domain are respectively shown in Figure 4b,c. One can learn that, because of the influence of heavy background noise, none obvious signal feature can be captured both in time domain and in frequency domain. The squared envelope spectrum (SES) of the simulation signal by conducting envelope analysis directly on the simulation signal is shown in Figure 4d, where we cannot recognize any transient fault features in the SES.

**Figure 5:**
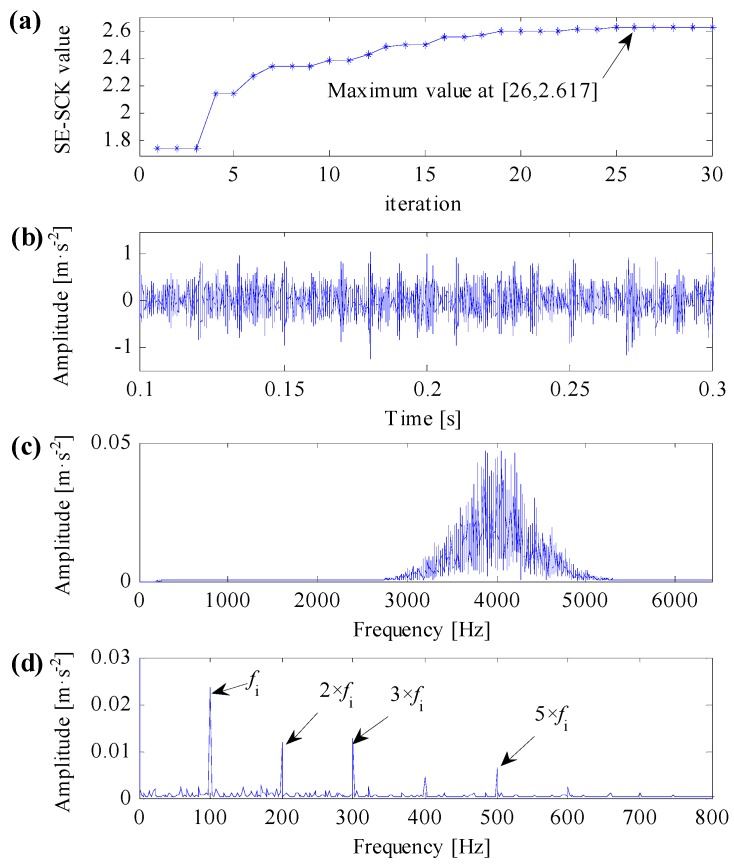
Analysis results of the proposed optimal resonant band demodulation based on the improved correlated kurtosis. (**a**) The iterative computation results of the PSO; (**b**) the filtered signal by optimal Morlet wavelet filter; (**c**) the spectrum of the filtered signal; (**d**) the SES of the filtered signal.

Firstly, utilizing the proposed optimal resonant band demodulation method to analyze the simulation signal, which is shown in Figure 4b. According to the algorithm flow in Figure 3, the impulsive feature frequency $f_{i}$ equals 100 Hz; the central frequency searching range of the optimal resonant band is set between 50 Hz and 6350 Hz; and the bandwidth searching range of the optimal resonant band is set between 100 Hz and 1000 Hz. By initializing the Particle Swarm Optimization algorithm, the calculation result after 30 times’ interactive computation is shown in Figure 5a, where the maximum of the improved correlated kurtosis occurs at 26th calculation, which equals 2.617, and the acquired optimal center frequency and bandwidth of resonant frequency band are 4015 Hz and 760 Hz, respectively.

Constructing the complex Morlet wavelet filter based on the optimal resonant frequency band parameters, the filtered simulation signal both in time domain and in frequency domain are shown in Figure 5b,c, respectively. The signal filtered by the optimal complex Morlet filter roughly shows transient impulse feature. The filtering central frequency of the optimal complex Morlet filter is almost identical to the resonant central frequency of the simulation signal, which equals to 4000 Hz, and the filtering frequency range can properly cover the resonant frequency band. The SES of the simulation signal performed by the proposed method is shown in Figure 5d, where the fault feature frequency $f_{i}$ and its harmonic frequencies are easy to identify. In the meanwhile, fault feature frequencies are dominant in frequency domain. Consequently, the improved correlated kurtosis can conquer the disturbance of impulsive noise, identify the repetitive transient impulse series. Combining complex Morlet filter and PSO optimization algorithm, the proposed optimal resonant band demodulation method can adaptively identify the optimal resonant band and demodulate fault features.

**Figure 6:**
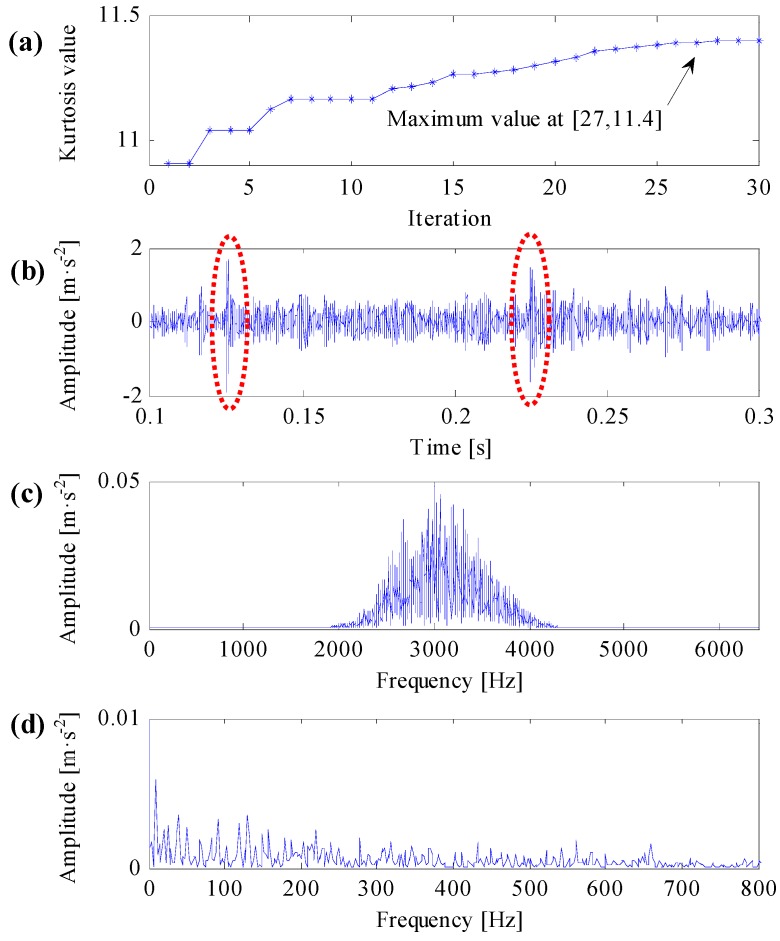
Analysis results of optimal resonant band demodulation based on kurtosis of the squared envelope signal. (**a**) The iterative computation results of the PSO; (**b**) the filtered signal by optimal Morlet wavelet filter; (**c**) the spectrum of the filtered signal; (**d**) the SES of the filtered signal.

**Figure 7:**
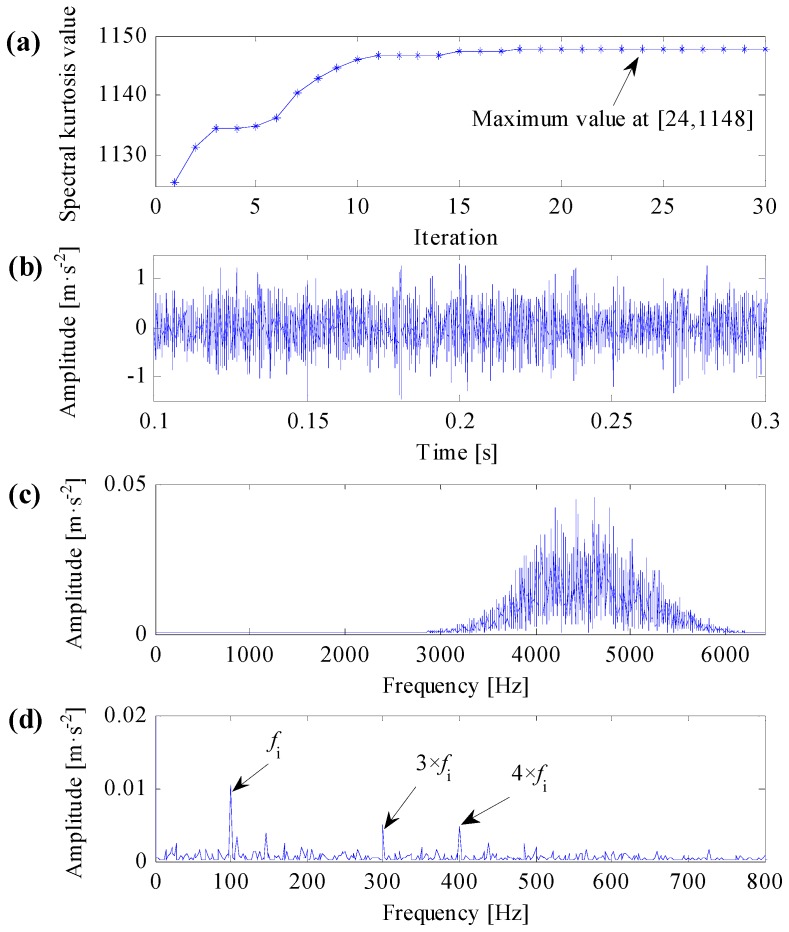
Analysis results of optimal resonant band demodulation based on spectral kurtosis of the squared envelope signal. (**a**) The iterative computation results of the PSO; (**b**) the filtered signal by optimal Morlet wavelet filter; (**c**) the spectrum of the filtered signal; (**d**) the SES of the filtered signal.

To compare the improved correlated kurtosis with kurtosis and spectral kurtosis, the kurtosis and spectral kurtosis of squared envelope signal are used to substitute the improved correlated kurtosis, respectively. According the same algorithm flow in Figure 3 and the same initialization parameters used in Figure 5, analysis results of the same simulation signal obtained by kurtosis and spectral kurtosis are shown in Figure 6 and Figure 7, respectively.

One can learn from Figure 6a that the maximal kurtosis of the squared envelope signal occurs at the 26th iterative calculation and the acquired optimal center frequency and bandwidth of resonant band are 3115 Hz and 723 Hz, respectively. Constructing complex Morlet wavelet filter based on the acquired optimal resonant frequency parameters, the filtered signal is shown in Figure 6b and its frequency spectrum shown in Figure 6c. From Figure 6b, it can be learned that, there are two prominent impulsive components in the filtered signal, which are consistent with the impulsive noise components. From Figure 6c, the filtering central frequency of the optimal complex Morlet filter is approaching to the resonant central frequency of the impulsive noise components, which equals to 2500 Hz. As a result, the filtered signal contains prominent impulsive noise components. From Figure 6d, the SES of the filtered signal cannot extract any distinct fault features.

One can learn from Figure 7a that the maximal spectral kurtosis of the squared envelope signal occurs at 24th iterative calculation and the acquired optimal center frequency and bandwidth of resonant band are 4560 Hz and 1000 Hz, respectively. Constructing complex Morlet wavelet filter based on the acquired optimal resonant frequency parameters, the filtered signal is shown in Figure 7b and its frequency spectrum is shown in Figure 7c. From Figure 7c, the filtering central frequency of the optimal complex Morlet filter is not identical to the resonant central frequency of the simulation signal, but the filtering frequency band still can properly cover the resonant central frequency of the simulation signal. Consequently, from Figure 7d, the SES of the filtered signal can extract distinct fault features.

As mentioned in this subsection, kurtosis can only characterize the strength of transient impulses, it cannot differentiate impulse noises and repetitive transient impulses, the SES of the filtered signal in Figure 6d cannot extract any useful fault features. The SES of the filtered signal in Figure 7d can extract correct fault features, but it contains smaller fault feature frequency and less harmonics. The SES of the filtered signal in Figure 5d can extract optimal fault features, which contains clear fault feature frequency and its harmonics. As a result, the improved correlated kurtosis can overcome shortcomings of kurtosis-based indexes, and the proposed optimal resonant band demodulation can identify the natural resonance frequency of rolling bearing vibration signals. Analysis results of the proposed method can clearly extract fault features and correctly diagnose rolling bearing faults, that is, the analysis results verify the validity and superiority of the proposed method.

### 4.2. Experimental Analysis

**Figure 8:**
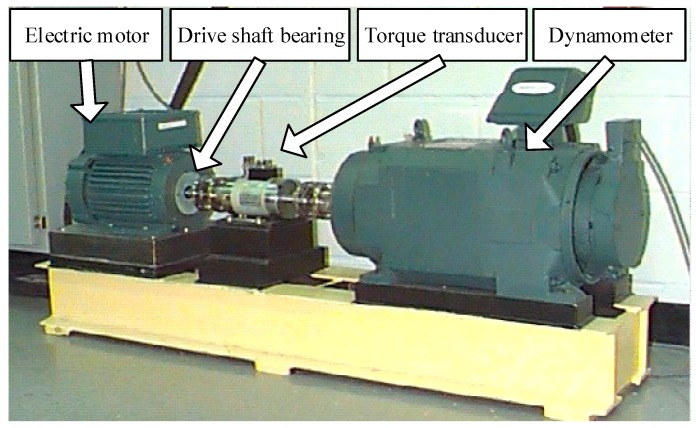
The bearing test rig of the CWRU Bearing Data Center [27].

Vibration data from the Case Western Reserve University (CWRU) Bearing Data Center are utilized to verify the validity of the proposed method. The bearing test rig of CWRU [27] is shown in Figure 8, which consists of a two horsepower Reliance Electric motors, a torque transducer/encoder, a dynamometer and control electronics. Motor bearings are seeded with faults using electro-discharge machining. Faults ranging from 0.007 inches to 0.021 inches in diameter are introduced separately on the inner ring. Faulty bearings are then reinstalled into the test rig with motor loads ranging from zero to three horsepower and motor speeds rotating at a rate between 1797 and 1720 RPM. Vibration data are collected using accelerometers, which are mounted at the 12 o’clock position at both the drive end and fan end of the motor housing. Vibration data sets are recorded using a data acquisition system and the sampling frequency is set to 12 KHz. According to the geometric parameters of the rolling bearing, the fault feature frequency of inner ring faults can be calculated as $f_{\mathrm{bpfi}}=5.415\times f_{r}$, where $f_{\mathrm{bpfi}}$ is the fault feature frequency and $f_{r}$ is the rotating frequency of the motor drive shaft. Data sampling conditions and their fault feature frequencies are listed in Table 3. Further details regarding the test setup can be found on the CWRU Bearing Data Center Website [27].

**Figure 9:**
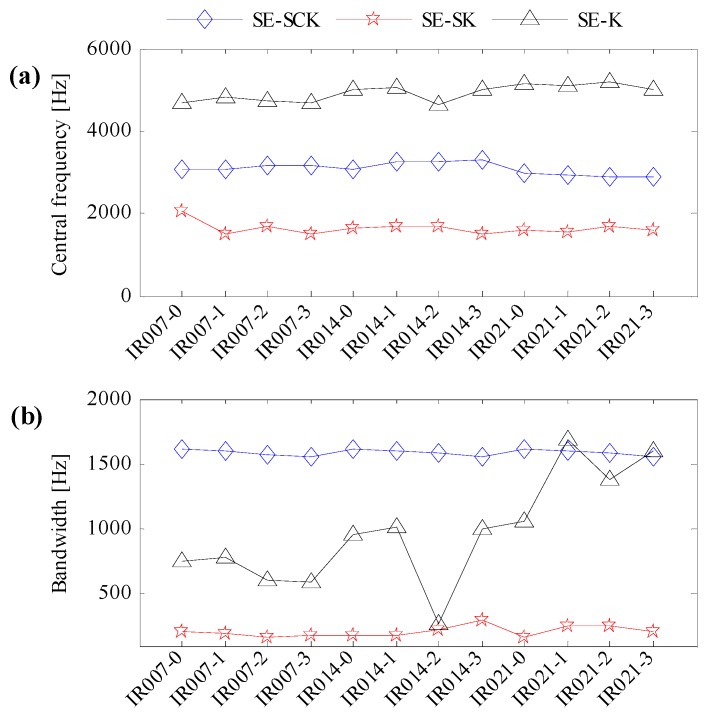
Identification results of optimal resonant frequency bands. (**a**) The optimal central frequencies; (**b**) the optimal bandwidth.

In line with the algorithm flow in Figure 3, three kinds of indexes, the improved correlated kurtosis (SE-SCK), the kurtosis of squared envelope signal (SE-K) and the spectral kurtosis of the squared envelope signal (SE-SK), are utilized to identify the optimal resonant frequency band of CWRU data. According to data sampling conditions and fault feature frequencies, the searching range of the optimal resonant frequency bandwidth is set as $f_{\mathrm{bpfi}}\leq \beta \leq 10\times f_{\mathrm{bpfi}}$; the searching range of the optimal resonant central frequency is set as $\beta_{\min}/2\leq f_{c}\leq f_{s}/2-\beta_{\min}/2$. Initializing the Particle Swarm Optimization algorithm and performing 30 times’ interactive computation. The identified resonant central frequency and bandwidth are shown in Figure 9a,b, respectively.

One can learn from Figure 9a,b that for the improved correlated kurtosis, the identified resonant central frequencies are centering around 3000 Hz, and the identified resonant frequency bandwidth is centering around 1600 Hz. For the kurtosis of squared envelope signal, the identified resonant central frequencies are around 5000 Hz, but the identified resonant frequency bandwidth is dispersed. For the spectral kurtosis of squared envelope signal, the identified resonant central frequencies are around 1500 Hz, and the identified resonant frequency bandwidth is around 160 Hz. Data IR007-0, which represents the minimal fault level and minimal motor load, and data IR021-3, which represents the maximal fault level and maximal motor load, are utilized to perform optimal resonant frequency band demodulation to verify the effectiveness of the identified resonant frequency bands in Figure 9.

**Figure 10:**
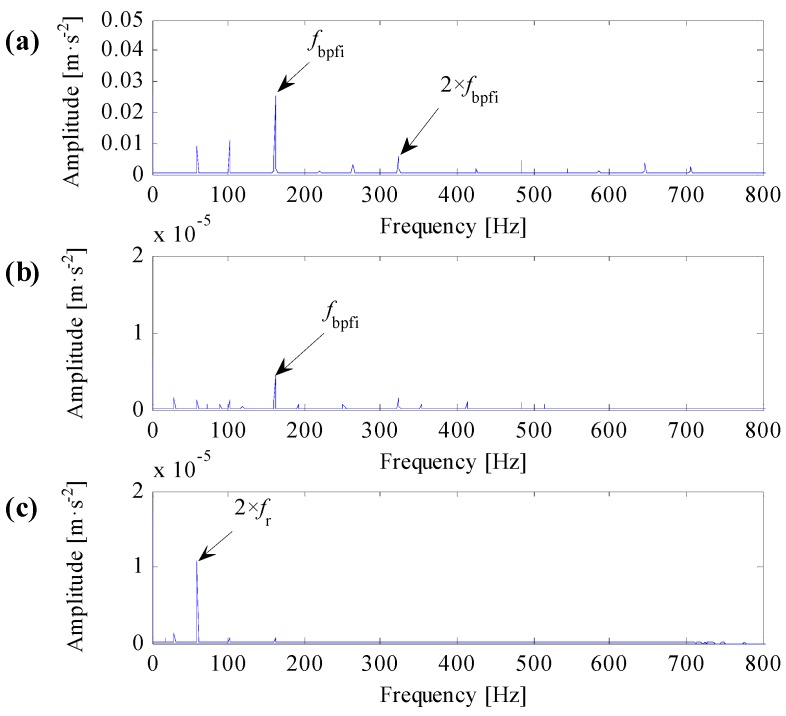
Analysis results of data IR007-0. (**a**) The result based on the improved correlated kurtosis; (**b**) the result based on the kurtosis of squared envelope signal; (**c**) the result based on the spectral kurtosis of squared envelope signal.

**Figure 11:**
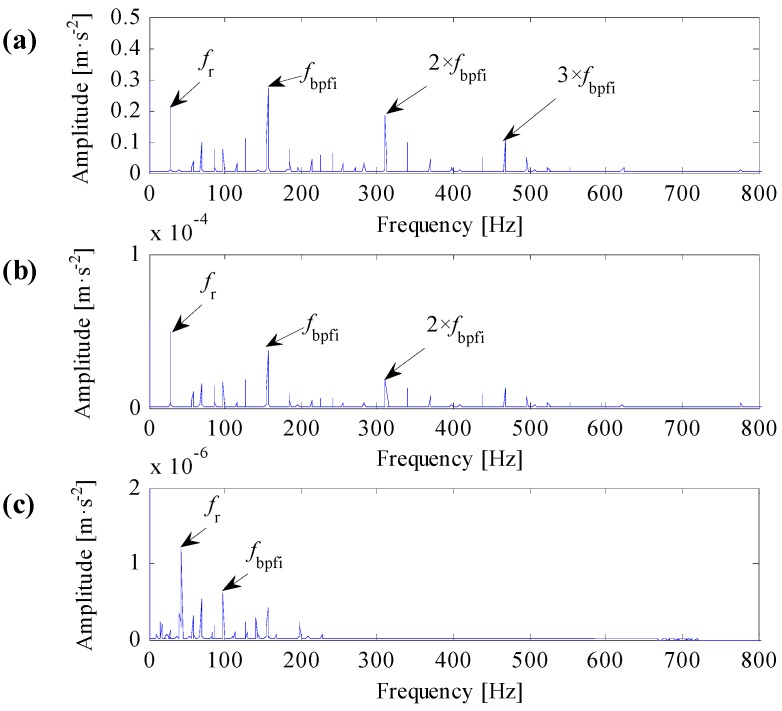
Analysis results of data IR021-3. (**a**) The result based on the improved correlated kurtosis; (**b**) the result based on the kurtosis of squared envelope signal; (**c**) the result based on the spectral kurtosis of squared envelope signal.

Analysis results of IR007-0 and IR021-3 are shown in Figure 10 and Figure 11, respectively.

One can learn from Figure 10a and Figure 11a that the identified resonant frequency bands based on the improved correlated kurtosis can steady extract fault feature frequency and its harmonics. The SES of optimal resonant frequency band filtered signals contain highlighted fault feature frequency, the value of fault feature frequency is higher than the value of rotating frequency, and fault feature frequency and its harmonics are dominant in frequency domain. As a result, the proposed optimal resonant frequency band demodulation based on the improved correlated kurtosis can clearly diagnose rolling bearing faults. From Figure 10b and Figure 11b, the identified resonant frequency bands based on the kurtosis of squared envelope signal can also recognize the fault feature frequency. However, for data IR007-0, harmonics of the fault feature frequency is too small to capture. For data IR021-3, values of fault feature frequencies and its harmonics are smaller than the value of rotating frequency, fault feature frequency and its harmonics are not dominant in frequency dominant. As a result, the optimal resonant frequency band demodulation based on the kurtosis of squared envelope signal cannot clearly diagnose rolling bearing faults. From Figure 10c and Figure 11c, the identified resonant frequency bands based on the spectral kurtosis can hardly recognize the fault feature frequency as for the extreme high value of rotating frequency. As a result, the optimal resonant frequency band demodulation based on the spectral kurtosis of squared envelope signal cannot clearly diagnose rolling bearing faults.

As is mentioned above, the proposed optimal resonant frequency band demodulation based on the improved correlated kurtosis can steady identify the optimal resonant bands, clearly demodulate fault feature frequencies and their harmonics, and effectively diagnose rolling bearing faults. Comparison analysis with kurtosis and spectral kurtosis have verified the superiority of the improved correlated kurtosis.

## 5. Conclusions

As to the drawbacks of kurtosis in detecting repetitive transient impulses and diagnosing rolling bearing faults, this manuscript firstly redefines correlated kurtosis based on auto-correlation function and the kurtosis. Then, it proposes an improved correlated kurtosis on the basis of squared envelope spectrum, which has been proved more effective in detecting repetitive transient impulses. Regarding the improved correlated kurtosis, it can not only characterize the strength of repetitive transient impulses, but also feature the cyclical occurrence of repetitive transient impulses, that is, the improved correlated kurtosis is effective in differentiating impulsive noises and repetitive transient impulses, and in reducing the noise impact on the detection of repetitive transient impulses as opposed to kurtosis-based indexes. Finally, this manuscript puts forward an optimal resonant band demodulation method based on improved correlated kurtosis by combining complex Morlet wavelet filter and the Particle Swarm Optimization algorithm. Analysis of both simulation signals and CWRU data demonstrate that, the proposed optimal demodulation method based on the improved correlated kurtosis can be more robust in identifying resonant frequency bands of rolling bearings and more accurate in demodulating transient fault features of rolling bearing. Analysis of data from experiment verify the validity and advantage of the proposed method over traditional kurtosis-based indexes.

## Figures and Tables

**Table 1:** The results of testing signals in time domain calculated by the kurtosis (K), the correlated kurtosis originated by McDonald (CK) and the redefined correlated kurtosis (ReCK).

Signal	K	CK	ReCK
**Y**_1_	15.0526	0	0
**Y**_2_	15.0526	0.2222	13.3333
**Y**_3_	15.0526	0.16	16
**Y**_4_	0.2928	0.0048	0.9694
**Y**_5_	−1.5	0.0068	1.35

**Table 2:** The results of testing signals in SES calculated by the spectral kurtosis (SK), and the improved correlated kurtosis (SESCK).

Signal	SK	SESCK
**Y**_1_	15.0526	0
**Y**_2_	15.0526	13.3333
**Y**_3_	15.0526	16

**Table 3:** Data sampling conditions and fault feature frequencies.

No.	Fault Depth/mm	Fault Diameter/mm	Motor Load/kW	Rotating Speed/rpm	Fault Feature Frequency/Hz
IR007-0	2.7940	0.1778	0	1797	162
IR007-1			0.7355	1772	159
IR007-2			1.4710	1748	157
IR007-3			2.2065	1721	155
IR014-0	2.7940	0.3556	0	1796	162
IR014-1			0.7355	1774	160
IR014-2			1.4710	1752	158
IR014-3			2.2065	1728	156
IR021-0	2.7940	0.5334	0	1797	162
IR021-1			0.7355	1774	160
IR021-2			1.4710	1752	158
IR021-3			2.2065	1728	156
